# A neonatal rat model of progressive left ventricular pressure overload induced by abdominal aortic banding microsurgery

**DOI:** 10.1016/j.xjtc.2025.04.014

**Published:** 2025-04-24

**Authors:** Zheng Wang, Sixie Zheng, Lincai Ye, Debao Li, He Zhang, Yingying Xiao, Chenxi Liu, Yuqing Hu, Sijuan Sun, Peisen Ruan, Hao Chen, Qi Sun

**Affiliations:** a Department of Thoracic and Cardiovascular Surgery, Shanghai Children's Medical Center, Shanghai Jiao Tong University School of Medicine, Shanghai, China; b Shanghai Institute For Pediatric Congenital Heart Disease, Shanghai Children's Medical Center, Shanghai Jiao Tong University School of Medicine, Shanghai, China; c Department of Pediatric Surgery, Children's Hospital of Fudan University, National Children's Medical Center, Shanghai, China; d Department of Critical Care Medicine, Women and Children's Hospital of Ningbo University, Ningbo, Zhejiang, China; e Department of Thoracic and Cardiovascular Surgery, Shanghai Children's Hospital, School of Medicine, Shanghai Jiao Tong University, Shanghai, China; f Department of Rheumatology and Immunology, Shanghai Children's Hospital, Shanghai Jiao Tong University School of Medicine, Shanghai, China; g Department of Cardiology, Shanghai Children's Hospital, School of Medicine, Shanghai Jiao Tong University, Shanghai, China; h Department of Pediatric Intensive Care Unit, Shanghai Children's Medical Center, Shanghai Jiao Tong University School of Medicine, Shanghai, China

**Keywords:** cardiomyocyte, left ventricular pressure overload, pediatrics, proliferation

## Abstract

**Objective:**

Left ventricular pressure overload models using adult mice or rats were developed 60 years ago; however, a neonatal mouse model of left ventricular pressure overload was reported only 5 years ago. Moreover, how left ventricular pressure overload reshapes the neonatal left ventricle and how it affects cardiomyocyte proliferation remain largely unexplored. The aim of this study is to develop a simple neonatal rat model with clinical features matched to those of left ventricular pressure overload.

**Methods:**

A neonatal rat model of progressive left ventricular pressure overload was created via abdominal aortic banding microsurgery at postnatal day 1 and verified by gross examination at postnatal day 7, abdominal ultrasound at postnatal day 21, and left upper limb blood pressure measurement from postoperative day 21 to day 35. A surgical video and detailed surgical procedures were documented for learning purposes.

**Results:**

RNA sequencing demonstrated that there were only 171 differentially expressed genes between the abdominal aortic banding surgery and sham left ventricles at postnatal day 3, with 406 differentially expressed genes at postnatal day 7. At postnatal day 3, there was little enrichment of proliferation-associated genes and only a small percentage of proliferating cardiomyocytes; at postnatal day 7, there was an abundant enrichment of proliferation-associated genes and a large percentage of proliferating cardiomyocytes, exactly opposite to the neonatal transverse aortic constriction surgery model, which exhibited decreased cardiomyocyte proliferation over time and even inhibited cardiomyocyte proliferation when severe left ventricular pressure overload was induced by transverse aortic constriction surgery. Moreover, abdominal aortic banding surgery does not require a thoracotomy, resulting in a success rate as high as 100%.

**Conclusions:**

A neonatal rat model of progressive left ventricular pressure overload was successfully established and fully documented to provide a platform for pediatric left ventricular pressure overload–associated investigation.

**Figure:**
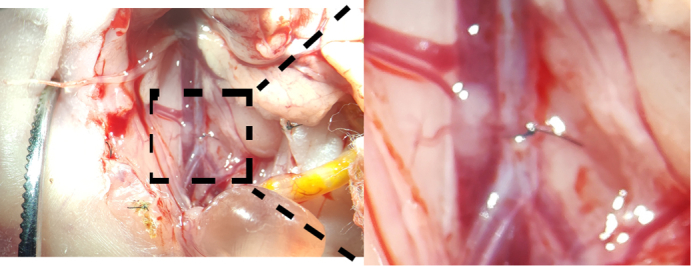
Neonatal ABS.

**Central Message**

A neonatal rat model of progressive LVPO via abdominal aortic banding microsurgery is introduced.

**Perspective**

LVPO is one of the most important features in children with cardiovascular diseases, such as aortic valve stenosis and hypertension. However, the current neonatal mouse and rat model of LVPO, established by TAC microsurgery, does not adequately recapitulate the features of pediatric cardiovascular diseases. We introduced a neonatal rat model of progressive LVPO via abdominal aortic banding microsurgery, providing a platform for understanding pediatric LVPO-associated diseases.

Left ventricular pressure overload (LVPO) is one of the most important features of various cardiovascular diseases, such as hypertension, aortic valve stenosis, and aortic valve calcification, in both children and adults.^1^, ^2^, ^3^ Thus, surgical LVPO models of adult mice and rats were developed 60 years ago, and they are currently used in thousands of studies, providing astonishing contributions to the understanding of LVPO-associated pathophysiology, such as cardiac hypertrophy and fibrosis.^4^, ^5^, ^6^ Currently, there are 2 types of LVPO surgical animal models: LVPO induced by transverse aortic constriction (TAC)^6^ surgery and LVPO induced by abdominal aortic banding surgery (ABS).^7^ Because the banding site of TAC is the ascending aorta, which is very close to the left ventricle (LV), the elastic aorta that would normally buffer the pressure caused by banding is lost, and therefore TAC induces a fixed LVPO with a rapid pathological progression of the LV. In contrast, the banding site of ABS is the abdominal aorta (AA), which is far from the LV, and therefore the elastic aorta is still available to buffer the pressure caused by banding. ABS induces a progressive LVPO and generates a slow pathological progression of the LV. Adult TAC animal models are more common than adult ABS animal models, and approximately 90% of studies reported in the literature used adult TAC to induce LVPO.

However, because of the challenges in constructing a surgical model of neonatal mice and rats (ice-bath anesthesia limiting the total surgical operation time to no more than 20 minutes, and the rice-sized small heart requiring the surgical operation to be performed under a microscope),^8^, ^9^, ^10^ a neonatal mouse model of LVPO induced by TAC was not introduced until 2019^11^ and was not fully described until 2021.^12^ In contrast, neonatal ABS was first reported 35 years ago.^13^ This is because a thoracotomy is a prerequisite for performing TAC, which is more difficult than performing ABS. Although neonatal ABS has been described for a long time and was also observed to induce cardiomyocyte (CM) proliferation, the detailed surgical protocols and videos were not documented.^7^^,^^13^^,^^14^ As a result, how LVPO remodels the neonatal LV, and particularly how it affects CM proliferation, remains largely explored.

Moreover, pediatric cases of hypertension, aortic valve stenosis, and aortic valve calcification induce a progressive LVPO, whereas TAC induces a fixed LVPO; therefore, the LVPO features of pediatric cardiovascular diseases and those of TAC are not well matched.^15^, ^16^, ^17^ It was reported that severe TAC did not promote CM proliferation and instead led to CM apoptosis, further supporting that TAC induced a fixed LVPO and a rapid pathological change of the LV.^18^ Neonatal TAC may well mimic pediatric coarctation of the aorta and interrupted aortic arch, both of which have a fixed LVPO,^19^^,^^20^ However, after surgical correction of coarctation of the aorta and interrupted aortic arch, children often develop postoperative aortic arch re-obstruction, LV outflow tract obstruction, or hypertension,^19^ which are characterized by a progressive LVPO. Thus, neonatal TAC models may not represent real clinical issues, and a neonatal model of progressive LVPO is needed.

The neonatal ABS model, performed on postnatal day 5, was first described in 1989 for studying LVPO-associated CM hypertrophy,^13^ and the neonatal ABS model, performed on postnatal day 2, was first described in 2003 for studying LVPO-associated CM proliferation.^21^ Moreover, ABS, but not TAC, induces hypertension.^22^ However, all previous neonatal ABS studies lacked the construction protocol, verification, and videos of neonatal ABS, making it difficult to master the neonatal ABS construction procedure; as a result, there are less than 10 reports of neonatal ABS as far as we know, whereas it is still largely unknown how progressive LVPO reshapes neonatal LV. In the current study, we performed neonatal ABS on postnatal day 1, providing a detailed neonatal ABS surgical protocol with a surgical video. Moreover, the bulk RNA-sequencing data of neonatal LVs under the influence of a progressive LVPO were documented for the first time. This study may help pave the way to understanding pediatric cardiovascular diseases with progressive LVPO.

## Material and Methods

### Statement of Data Availability

RNA-sequencing data were deposited in the National Center for Biotechnology Information's Gene Expression Omnibus database (https://www.ncbi.nlm.nih.gov/geo), with accession number GSE289819.

### Ethics Statement

All procedures described in this study were approved by the Animal Welfare and Human Studies Committee of Shanghai Children's Medical Center (Institutional Review Board approval No. SCMC-LAWEC-2024-1127, November 24, 2024).

### Abdominal Aortic Banding Surgery

The neonatal ABS comprised 3 steps: preoperative preparation, ABS, and postoperative care.

#### Preoperative preparation

**Figure E1:**
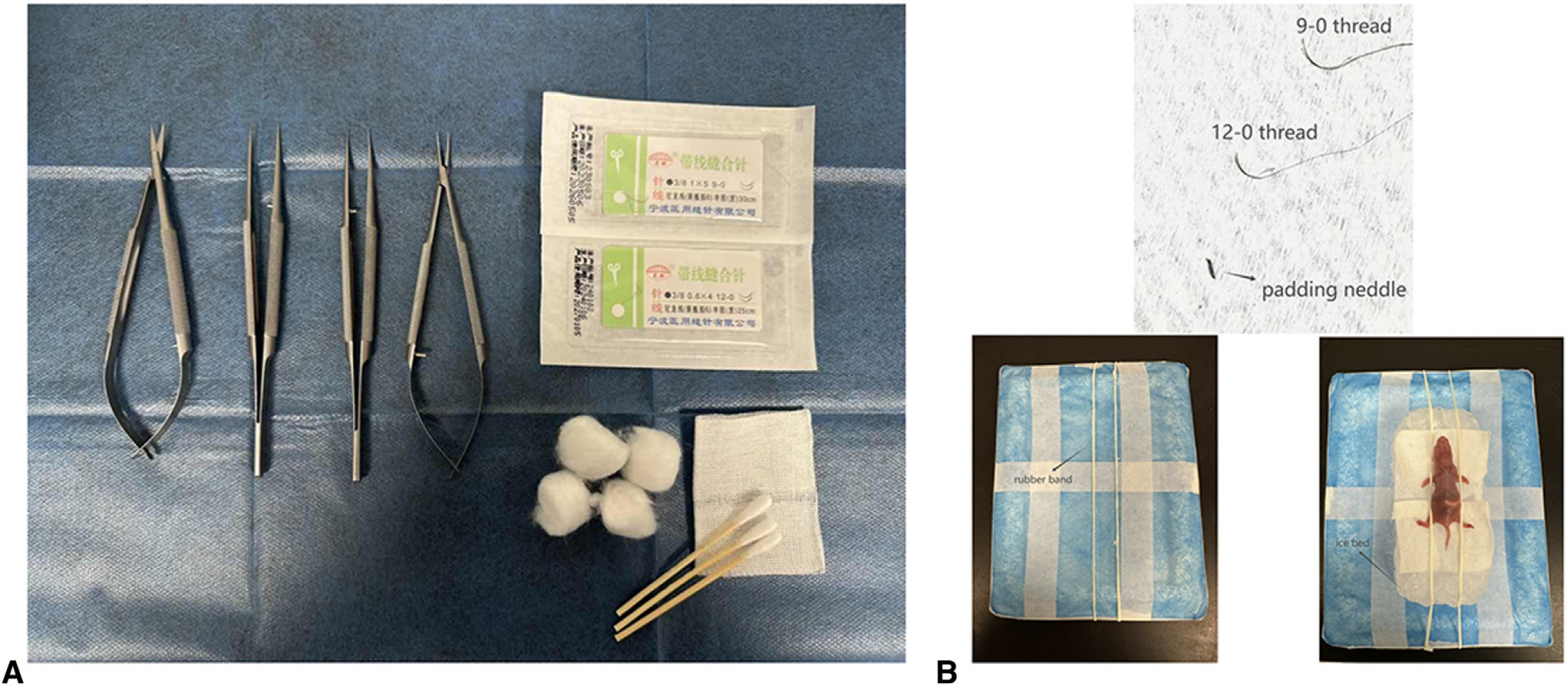
The surgical tools and materials for neonatal ABS. A, Surgical Items. B, Self-made tools required for neonatal ABS include padding needle, rubble band, and ice bed for operation. *ABS*, Abdominal aortic banding surgery.

Several surgical items were prepared in advance, including 30G padding needles, tiny hemostatic gelatin sponges, 9/12-0 thread with a blunt needle tip, and a water-resistant ice bed. The 30G padding needle was cut to a suitable size so that it could be placed into the small abdominal cavity. The tip of the 12-0 thread needle was ground to a blunt end to avoid pricking blood vessels. The ice bed was made using a Styrofoam box and was enclosed by a sterile towel. A concave position at the center of the ice bed was used to store ice. Two parallel rubber bands were used for rat fixation. Self-made surgical instruments were sterilized before surgery to reduce the risk of infection. The surgical items and instruments are presented in Figure E1.

#### Neonatal abdominal aortic banding surgery

Sprague–Dawley pups bred in our laboratory, male and female, were anesthetized by direct ice cooling for approximately 3 to 5 minutes. The anesthetized neonates were then transferred to an ice bed and fixed in the supine position. A longitudinal incision of approximately 1.5 to 2 cm was made at the lower midline of the abdomen, and the skin, subcutaneous tissue, and abdominal muscles were cut layer-by-layer to expose the abdominal cavity. the detailed protocols are described in the “Results” section.

#### Postoperative care

The pups were warmed under a heat lamp set to 40 °C until they were breathing freely (2-3 minutes) and then transferred to another heat lamp set to 37 °C until they were able to make natural movements and their skin turned red or pink (2-3 hours). All the pups were returned together as a group to their mother.

### Study Design

A total of 48 rat pups (24 per experimental group) were used in this study, with specific allocation details provided in Table E1. Twelve rats (6 per group) designated for serial blood pressure (BP) measurements and abdominal ultrasound monitoring survived through postoperative day 35 before undergoing euthanasia. Cardiac tissues from these subjects were cryopreserved at −80 °C in ultra-low temperature freezers for subsequent analysis. Although neonatal ABS presents significantly lower technical demands compared with neonatal TAC, a learning curve exists for surgical proficiency. Inexperienced operators may consequently encounter elevated mortality rates during the initial phase of technique acquisition.

### Abdominal Ultrasound

At postnatal day 21, rats were anesthetized with isoflurane and allowed to breathe spontaneously through a nasal cone (isoflurane/oxygen: 1.5%-2.0% maintenance). The banding site was analyzed using a Vevo 2100 imaging system (Visual Sonics). To confirm stenosis of the AA, the velocity time integral of the AA blood flow was calculated from the mean of 3 consecutive measurements using 2-dimensional and pulse Doppler echocardiography.

### Blood Pressure Measurement

Systolic blood pressure (SBP) and diastolic blood pressure (DBP) were measured in conscious, restrained rats using a noninvasive tail-cuff plethysmography system (IITC Life Science MRBP BP system) calibrated for monitoring left upper limb artery. Measurements were acquired at postoperative day 21 to day 35 at fixed times. The mean arterial pressure was then calculated (mean arterial pressure = (2 (DBP) + SBP)/3).

### Immunofluorescence

At postnatal days 3 and 7, the hearts were harvested, sectioned, and subjected to immunofluorescences staining. Briefly, tissue sections were washed 3 times with phosphate-buffered saline, fixed in 4% paraformaldehyde, permeabilized with 0.5% Triton X-100, and then incubated with anti-Ki67 (ab15580, Abcam), anti-Aurora B (ab2254, Abcam), and anti-cardiac troponin T (ab8295, Abcam) antibodies overnight at 4 °C. The slides were then incubated with secondary antibodies and DAPI, photographed under a confocal microscope, and analyzed with ImageJ software (National Institutes of Health).

### RNA Sequencing

At postnatal days 3 and 7, the LVs were collected for RNA sequencing. Sequencing libraries were generated with a NEBNext Ultra RNA library prep kit for Illumina according to the manufacturer's recommendations. The library quality was assessed on an Agilent Bioanalyzer 2100 system. Library sequencing was performed on an Illumina NovaSeq platform. Clean data (clean reads) were generated by removing reads that contained adapters, low-quality reads, and reads containing poly-N from the raw data. All subsequent analyses used high-quality clean data. Feature Counts v1.5.0-p3 was used to determine the number of reads. The fragments per kilobase of transcript sequence per million base pairs sequenced of each gene was calculated. Differential expression analysis was conducted using the DESeq2 R package (1.16.1). We adjusted the resulting *P* values with Benjamini and Hochberg's approach to control the false discovery rate. Genes with an adjusted *P* less than .05 detected by DESeq2 were considered differentially expressed. An enrichment analysis was conducted using the clusterProfiler R package, with a corrected *P* less than .05 considered as significantly enriched.

### Statistical Analysis

Continuous data were expressed as mean ± SD. We tested differences using the Student *t* test, 1-way analysis of variance, and SNK when the data were normally distributed; otherwise, differences were assessed using the rank-sum test. Statistical analyses were conducted using SAS software v. 12.0 (SAS Institute Inc).

## Results

### Neonatal Abdominal Aortic Banding Microsurgery

**Figure 1:**
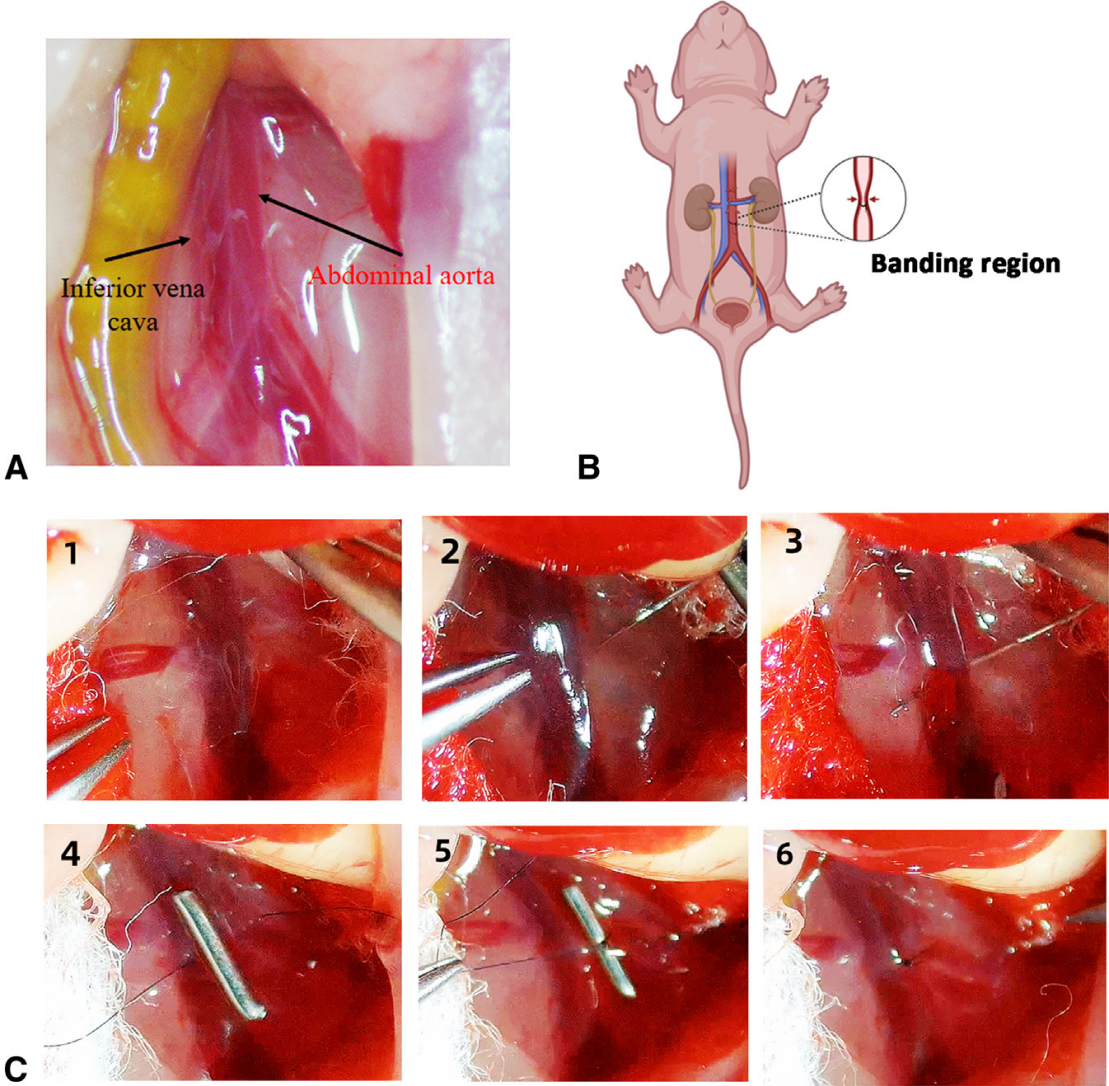
ABS procedure. A, Local anatomy at the site of the ABS. B, Schematic diagram of ABS. C, Key steps of ABS. C1, Expose the abdominal aorta. C2 and C3, A 12-0 suture needle was passed through the AA. The needle was held with a needle holder on the opposite side as it was passed through and then gently pulled until the suture thread was completely under the AA. C4 and C5, The padding needle was placed on the AA, and the AA and padding needle were ligated with 12-0 thread. Then, the padding needle was gently removed using a micro holder to create a lumen with a diameter equal to that of the needle. C6, The ends of the sutures were cut. *ABS*, Abdominal aortic banding surgery; *AA*, abdominal aorta.

**Figure E2:**
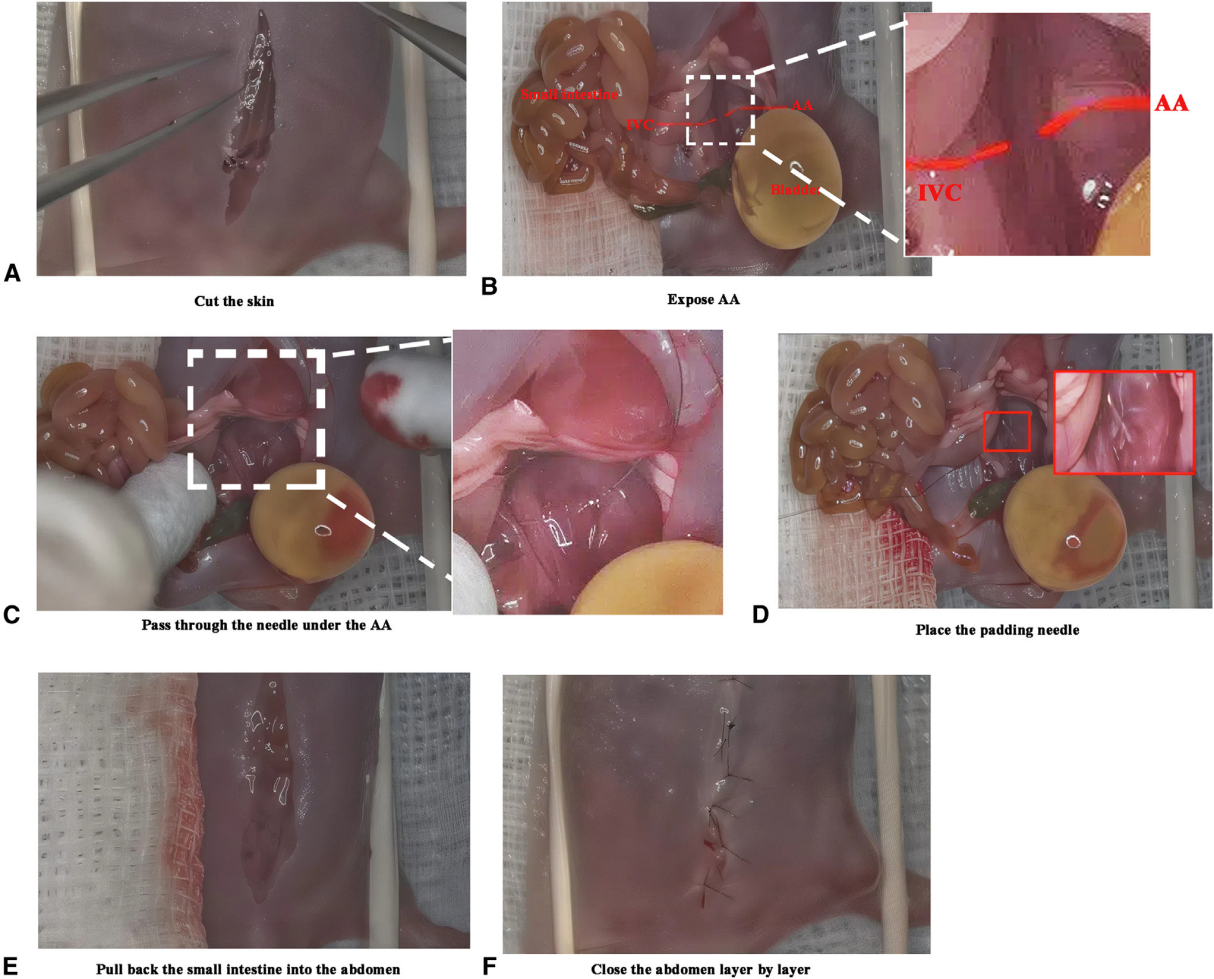
ABS process. A, Longitudinal incision of approximately 1.5 to 2 cm was made at the lower midline of the abdomen. B, The small intestine was pulled to expose the AA. C, The suture needle was passed under the AA. D, The padding needle was placed and ligated. E, The small intestine was placed back in the abdomen. F, The abdomen was closed layer-by-layer. *AA*, Abdominal aorta; *IVC*, inferior vena cava.

As shown in Video 1, the skin was carefully lifted with tweezers while cutting open the skin with scissors under a microscope (Figure E2, *A*), and then the small intestine was pulled out of the abdomen with a cotton swab, clearly exposing the AA (Figure 1, *A* and *B*, and Figure E2, *B*). Pulling the small intestine with tweezers was cautiously avoided, because this can easily cause the small intestine to rupture and bleed. A 12-0 suture needle was passed through the AA (Figure 1, *C*1-3 and Figure E2, *C*). The needle was held with a needle holder on the opposite side as it was passed through and then gently pulled until the suture thread was completely under the AA (Figure 1, *C*4). The padding needle was placed on the AA, and the AA and padding needle were ligated with 12-0 thread (Figure 1, *C*4-6 and Figure E2, *D*). Then, the padding needle was gently removed using a micro holder to create a lumen with a diameter equal to that of the needle (Figure 1, *C*6). The ends of the sutures were cut. The small intestine was carefully placed back into the abdomen with a cotton swab (Figure E2, *E*) while avoiding rupture and bleeding of the small intestine. Finally, the abdomen was closed layer-by-layer (Figure E2, *F*). The pups were warmed under a heating lamp set to 37 °C until they could breathe freely (2-3 minutes). The pups were then transferred to another heating lamp set to 37 °C until they made natural movements and exhibited red or pink skin (2-3 hours). The survival was 100%.

### Abdominal Aortic Banding Surgery Induced a Progressive Left Ventricular Pressure Overload

**Figure 2:**
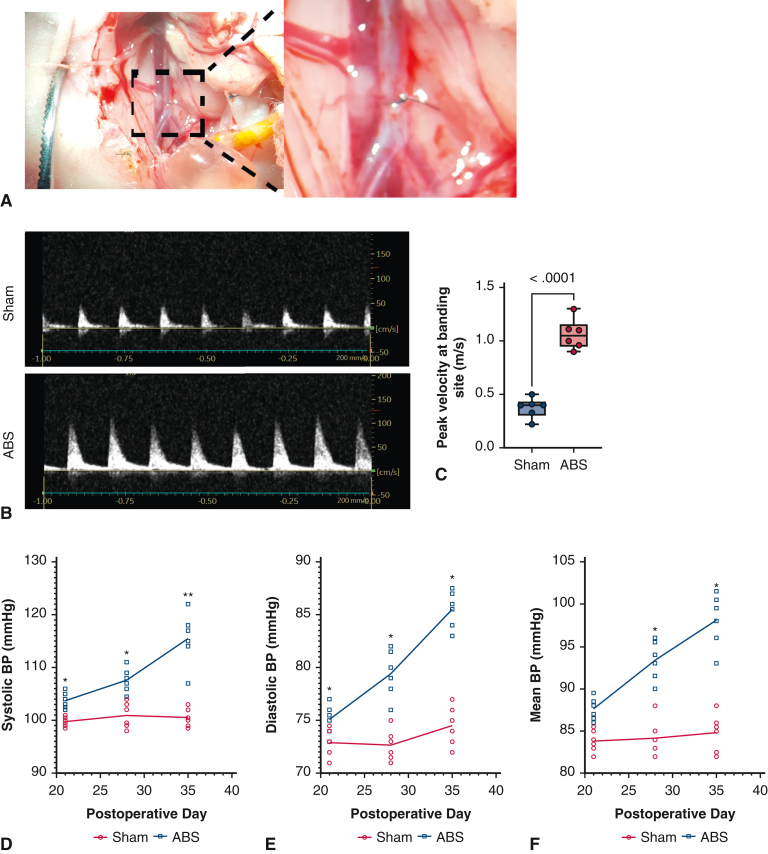
Verification of LVPO. A, At postnatal day (P)7, a banding suture around the AA was visible under a microscope. B, Representative abdominal ultrasonography at the banding site at P21. C, Quantification of peak velocity at the banding site. The lower and upper borders of the box represent the lower and upper quartiles (25th percentile and 75th percentile). The *middle horizontal line* represents the median. The lower and upper whiskers represent the minimum and maximum values of nonoutliers. N = 6 rats. D, Systolic BP increased over time from postoperative day 21 to 35 because of the ABS. E, Diastolic BP increased over time from postoperative day 21 to 35 because of the ABS. F, Mean BP increased over time from postoperative day 21 to 35 because of the ABS. *LVPO*, Left ventricular pressure overload; *AA*, abdominal aorta; *ABS*, abdominal aortic banding surgery. ∗*P* < .05; ∗∗*P* < .01.

**Figure E3:**
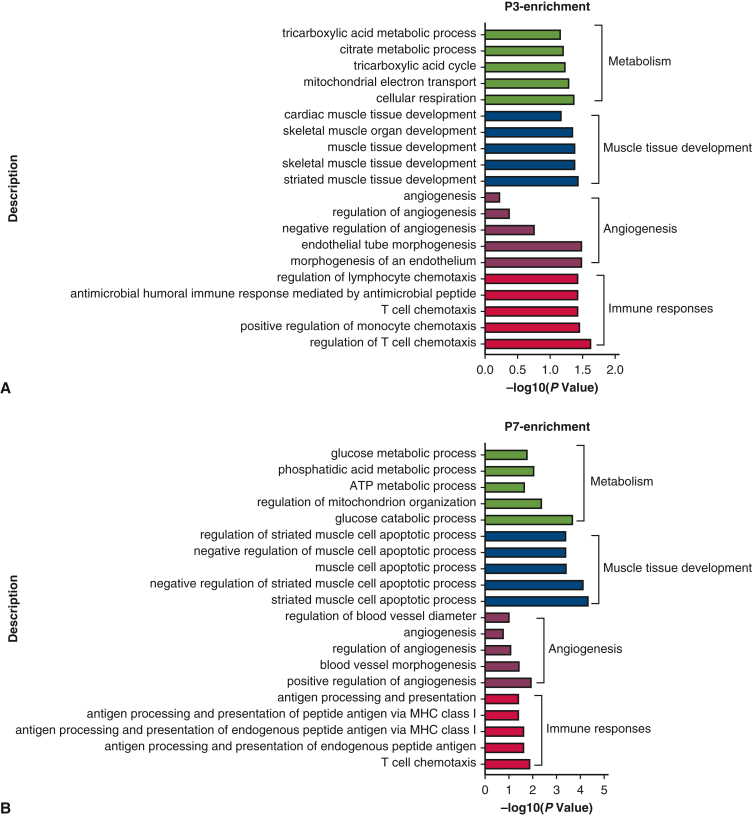
Key biological terms enriched by ABS at different time points. A, Enriched GO terms at P3. B, Enriched GO terms at P7. *ABS*, Abdominal aortic banding surgery.

As shown in Figure 2, *A* and *B*, at postnatal day 7, a thread-fixed AA was clearly visible under a microscope. Abdominal ultrasound showed a significant increase in the blood flow velocity at the banding site in the ABS group compared with that of the sham group at postnatal day 21 (Figure 2, *C* and *D*). Because of the small body size of neonatal rats, a rat tail BP measurement device was unable to detect the BP during the first 2 postoperative weeks. Therefore, we chose to monitor BP from the third postoperative week. The results showed that from postoperative day 21 to day 35, there was a continuous and slow increase in the systolic, diastolic, and mean BP in the ABS group but not in the sham group (Figure 2, *E* and *F*). In addition, at postoperative day 35, a significant increase of LV thickness was observed in the ABS group (Figure E3). These results suggested that ABS induced a progressive LVPO.

### The Gradually Increasing Number of Differentially Expressed Genes From Postnatal Day 3 to 7 Suggests a Progressive Increase in Left Ventricular Pressure Overload

**Figure 3:**
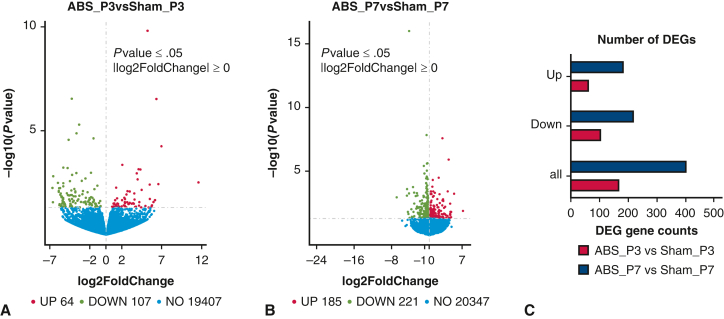
A progressive LVPO indicated by a gradually increasing number of DEGs from P3 to P7. A, Volcano plot of DEGs at P3. B, Volcano plot of DEGs at P7. C, Quantification of DEGs at P3 and P7. *LVPO*, Left ventricular pressure overload; *DEG*, differentially expressed gene; *ABS*, abdominal aortic banding surgery.

As shown in Figure 3, *A*, there were only 171 differentially expressed genes (DEGs) between the ABS and sham groups at postnatal day 3, with 64 upregulated and 107 downregulated when comparing the ABS group with the sham group. At postnatal day 7, the number of DEGs increased more than 2-fold to 406, with 185 upregulated and 221 downregulated when comparing the ABS group with the sham group (Figure 3, *B*). These results suggested that the number of DEGs gradually increased over time during the neonatal stage (from postnatal day 1 to 7) (Figure 3, *C*), indicating a progressive increase in LVPO.

### High Reproducibility of Abdominal Aortic Banding Surgery at the Transcriptome Level

**Figure 4:**
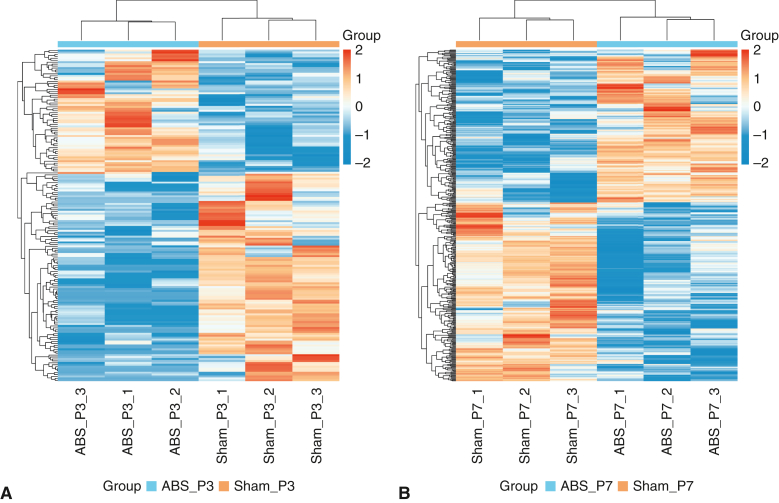
High reproducibility of ABS at the transcriptome level. A, Cluster analysis of DEGs at P3. B, Cluster analysis of DEGs at P7. *ABS*, Abdominal aortic banding surgery; *DEG*, differentially expressed gene.

A cluster analysis of DEGs showed that there were high inter-group differences and intra-group consistencies at postnatal days 3 and 7 after ABS or sham surgery (Figure 4, *A* and *B*). Principal component analysis also showed that the differences between groups were significant, whereas the differences within groups were relatively small at postnatal days 3 and 7 after ABS or sham surgery (Figure 4). These results suggested there was a highly reproducibility of ABS at the transcriptome level.

### More Abundant Cell Proliferation-Associated Enrichment at Postnatal Day 7 Than at Postnatal Day 3

**Figure 5:**
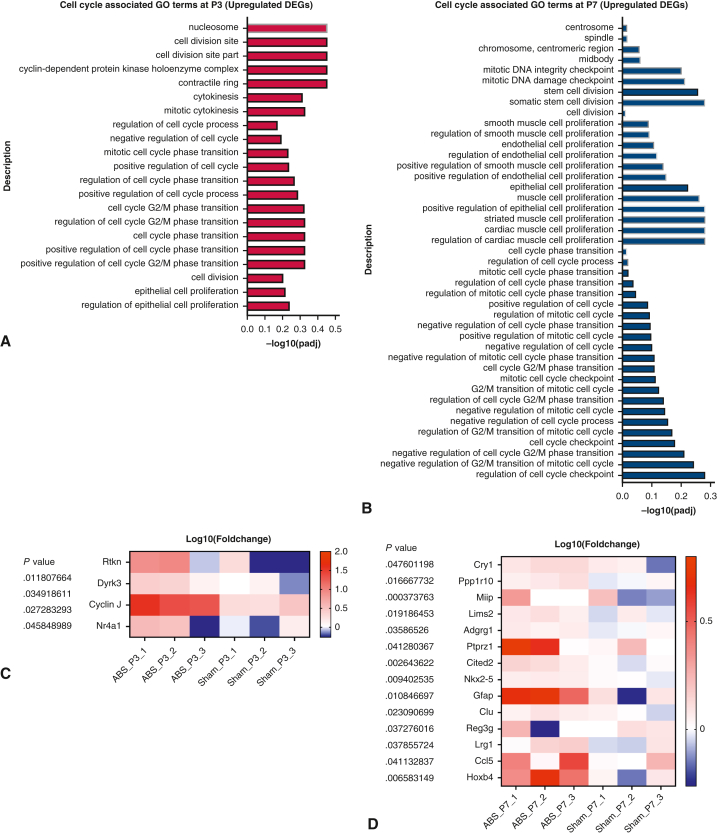
Gene ontology (*GO*) analysis of upregulated DEGs. A, Cell proliferation-associated GO terms at P3. B, Cell proliferation–associated GO terms at P7. C, Heatmap of genes associated with cell proliferation at P3. D, Heatmap of genes associated with cell proliferation at P7. *DEG*, Differentially expressed gene.

Gene ontology enrichment analysis of upregulated DEGs revealed that at postnatal day 3, of the 699 enrichment terms, there were 21 terms associated with cell proliferation (Figure 5, *A*), whereas at postnatal day 7, of the 2635 enrichment terms, there were 44 terms associated with cell proliferation (Figure 5, *B*). There were more upregulated genes associated with cell proliferation at postnatal day 7 than at postnatal day 3 (Figure 5, *C* and *D*). These results further confirmed that neonatal ABS induced a progressive increase in LVPO, characterized by increased cell cycle activities at the neonatal stage.

### More Cell Cycle-Active Cardiomyocytes at Postnatal Day 7 Than at Postnatal Day 3

**Figure 6:**
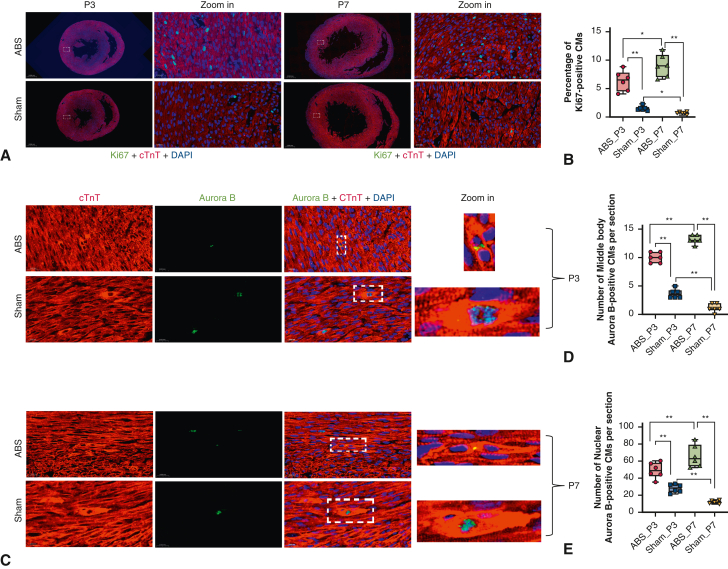
More CMs were active in the cell cycle at P7 than at P3. A, Representative Ki67-positive CMs at P3 and P7. Ki67 (*green*); cTnT (*red*); DAPI (*blue*). B, Quantification of Ki67-positive CMs. C, Representative Aurora B-positive CMs at P3 and P7. D, Quantification of middle body Aurora B-positive CMs. E, Quantification of nuclear Aurora B-positive CMs. The lower and upper borders of the box represent the lower and upper quartiles (25th percentile and 75th percentile). The *middle horizontal line* represents the median. The lower and upper whiskers represent the minimum and maximum values of nonoutliers. N = 6 rats. *CM*, Cardiomyocyte; *ABS*, abdominal aortic banding surgery.

To confirm the RNA-sequencing results, we examined the cell cycle markers Ki67 and Aurora B. The results showed that at postnatal day 3, compared with the sham group, the percentage of Ki67-positive CMs in the ABS group increased 4.1-fold, whereas at postnatal day 7, the percentage of Ki67-positive CMs in the ABS group increased 12.5-fold (Figure 6, *A* and *B*), indicating that there were still more Ki67-positive CMs in the ABS group at postnatal day 7 than in the ABS group at postnatal day 3 (Figure 6, *A* and *B*). Likewise, the number of Aurora B-positive CMs per section in the ABS group was significantly greater than in the sham group (Figure 6, *C-E*), and there were more Aurora B-positive CMs per section in the ABS group at postnatal day 7 than in the ABS group at postnatal day 3 (Figure 6, *C-E*).

## Discussion

LVPO is one of the most important features of many cardiovascular diseases, both in adults and children.^1^^,^^2^ Because of the ease of constructing surgical models in adult mice and rats, adult LVPO models were introduced earlier than neonatal LVPO models; therefore, how LVPO reshapes the adult LV, such as through cardiac hypertrophy and fibrosis, is clear, and many targets for treating cardiac hypertrophy and fibrosis have been proposed.^22^^,^^23^ In contrast, the construction of a surgical model of neonatal mice and rats is challenging; consequently, a neonatal LVPO model induced by TAC was introduced only recently.^11^^,^^12^ Although the neonatal ABS rat model was first reported 35 years ago, no detailed neonatal ABS construction protocols have been documented. As a result, little is known about the mechanisms by which LVPO reshapes the neonatal LV, and no methods have been reported for improving neonatal LV performance, particularly CM proliferation. It has been reported that neonatal hearts can fully adapt to LVPO-associated maladaptations without cardiac fibrosis through CM proliferation.^11^ Thus, understanding how LVPO reshapes the neonatal LV, and in particular how it affects CM proliferation, will reduce both pediatric and adult LVPO-associated maladaptation. This study is the first to document a neonatal ABS construction protocol. To enhance methodological reproducibility, we implemented rigorous surgical standardization encompassing 2 critical domains: (1) procedural parameters (banding size determined by banding needle, banding position determined by precise anatomic landmarks); and (2) instrumentation specifications (9-0 polypropylene sutures and a custom-designed padding needle). The ABS technique was systematically documented through high-resolution intraoperative imaging (Video 1) complemented by schematics (Figure 1 and Figure E2) detailing (1) topographical anatomic landmarks for band placement and (2) band position. This multimodal documentation framework represents a distinct methodological advancement over conventional surgical reports in neonatal ABS models. This study introduced an important platform for understanding how LVPO reshapes neonatal LVs and providing a relatively simple means by which learners can master neonatal ABS construction techniques.

**Figure E4:**
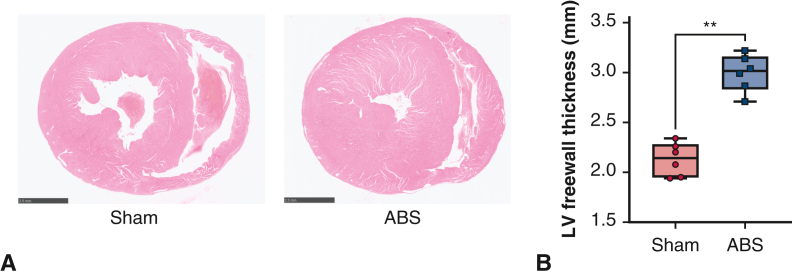
ABS induces an increase of LV thickness on postoperative day 35. A, Representative hematoxylin–eosin staining. B, Quantification of LV thickness. The lower and upper borders of the box represent the lower and upper quartiles (25th percentile and 75th percentile). The *middle horizontal line* represents the median. The lower and upper whiskers represent the minimum and maximum values of nonoutliers. N = 6 rats. *ABS*, Abdominal aortic banding surgery.

Compared with neonatal TAC, neonatal ABS has at least 2 advantages. First, LVPO induced by neonatal ABS is a progressive LVPO, which is consistent with most clinical diseases.^15^, ^16^, ^17^ In contrast, TAC induces a fixed LVPO. Second, thoracotomy is a prerequisite for performing TAC, rendering the procedure more difficult and resulting in a lower survival than ABS. When considering transcriptomic changes, neonatal ABS (postnatal day 3 or 7) and mild TAC shared a degree of common enrichment, including cell proliferation, angiogenesis, immune response, and muscle tissue development enrichment^11^ (Figures 5 and E4). However, neonatal ABS also induced metabolic changes (Figure E4), which were not found in neonatal TAC.^11^ The summary of key differences between ABS and TAC is shown in Table E2. Compared with ABS at postnatal day 3, ABS at postnatal day 7 induced a greater abundance of terms associated with cell proliferation, angiogenesis, immune responses, and muscle tissue development (Figures 5 and E4), further confirming that from postnatal day 1 to 7, there was progressive LVPO at the transcriptomic level.

Immune responses and metabolic changes associated with CM proliferation are now well documented.^24^^,^^25^ However, whether the immune response and metabolic changes are the cause of CM proliferation or the result of CM proliferation still needs further clarification.^26^ Furthermore, whether neonatal LVPO induces a unique regulatory mechanism of CM proliferation should be the focus of further investigation. In addition, there are currently no reports of neonatal mouse models of ABS, as far as we know. Considering that many genetically modified animals are mice and not rats, another research focus in the future should be to successfully construct a neonatal mouse model of ABS. However, it should be noted that, as shown in Figure 1, *A*, the range of the neonatal rat AA that can be used for ABS is very small, let alone the neonatal mouse AA, with a greatly limited surgical space.

## Conclusions

Neonatal ABS is an experimental model that introduces LVPO during early postnatal development. This model provides unique insights into the cardiac response to increased afterload in the context of a developing heart, which differs significantly from the adult heart in terms of cellular plasticity, regenerative capacity, and adaptive mechanisms. The key aspects of neonatal ABS and the new information it brings to the clinical context are summarized in Table E3.

## Conflict of Interest Statement

The authors reported no conflicts of interest.

The *Journal* policy requires editors and reviewers to disclose conflicts of interest and to decline handling or reviewing manuscripts for which they may have a conflict of interest. The editors and reviewers of this article have no conflicts of interest.

## Figures and Tables

**Table E1:** Survival and experimental allocation

Group	RNA sequencing P3	RNA sequencing P7	IF-P3	IF-P7	BP/ultrasound
Sham	3	3	6	6	6
ABS	3	3	6	6	6

*P*, Postnatal day; *IF*, immunofluorescence; *BP*, blood pressure; *ABS*, abdominal aortic banding surgery.

**Table E2:** Summary of key differences between abdominal aortic banding surgery and transverse aortic constriction surgery

Surgery	ABS	TAC
Location	Abdominal aorta (below renal arteries)	Transverse aortic arch
Pressure overload	Gradual and chronic	Acute and severe
Hypertrophy	Gradual, compensatory	Rapid, often maladaptive
Hyperplasia	CM hyperplasia (an increase in cell number) is not typically observed in adult mammalian. In neonatal hearts, CM hyperplasia increases with time.	CM hyperplasia (an increase in cell number) is not typically observed in adult mammalian. In neonatal hearts, CM hyperplasia decreases with time, and on severe constriction condition, CM hyperplasia may not occur, but rather CM apoptosis and necrosis.
Fibrosis	Slower progression, less severe	Rapid and pronounced
Functional outcome	HFpEF (diastolic dysfunction)	HFrEF (systolic and diastolic dysfunction)
Clinical relevance	Chronic hypertension, coarctation	Acute aortic stenosis, sudden afterload

*ABS*, Abdominal aortic banding surgery; *TAC*, transverse aortic constriction; *CM*, cardiomyocyte; *HFpEF*, heart failure with preserved ejection fraction; *HFrEF*, heart failure with reduced ejection fraction.

**Table E3:** Clinical and mechanistic insights from the neonatal abdominal aortic banding surgery model

Key aspects of neonatal ABS	Neonatal hearts	Clinical relevance
CM hyperplasia vs hypertrophy	◆ Neonatal CMs retain the ability to proliferate (hyperplasia) in response to stress, unlike adult CMs, which primarily undergo hypertrophy (increase in cell size). ◆ Neonatal ABS can induce both hyperplasia and hypertrophy, providing a model to study how the heart balances these 2 responses to pressure overload.	◆ Understanding the mechanisms of neonatal CM proliferation could inform strategies to promote cardiac regeneration in adults, such as after myocardial infarction or in heart failure. ◆ This model may help identify molecular pathways that can be reactivated in adult hearts to induce hyperplasia instead of hypertrophy, potentially preventing maladaptive remodeling.
Enhanced regenerative capacity	◆ The neonatal heart has a remarkable capacity for regeneration, which diminishes rapidly after the first wk of life in rodents and shortly after birth in humans. ◆ Neonatal ABS can be used to study how pressure overload impacts this regenerative capacity and whether it can be harnessed to repair the heart.	◆ Insights from neonatal ABS could lead to therapies that enhance regenerative capacity in pediatric patients with congenital heart defects or acquired heart disease. ◆ This model may also help identify factors that limit regeneration in adults, paving the way for regenerative medicine approaches.
Adaptive vs maladaptive remodeling	◆ Neonatal ABS often results in adaptive remodeling, with preserved systolic function and minimal fibrosis, unlike adult models where pressure overload typically leads to maladaptive remodeling and heart failure. ◆ The neonatal heart may activate unique protective mechanisms, such as increased angiogenesis or altered metabolic pathways, to cope with pressure overload.	◆ Identifying these protective mechanisms could lead to novel therapeutic targets for preventing or reversing maladaptive remodeling in adults. ◆ This model may help explain why some pediatric patients with congenital heart disease (eg, aortic coarctation) tolerate pressure overload better than adults with similar conditions.
Developmental plasticity	◆ The neonatal heart is highly plastic, meaning it can adapt its structure and function in response to changes in hemodynamic load. ◆ Neonatal ABS can induce changes in ventricular geometry, wall thickness, and chamber size, providing insights into how the heart remodels during development.	◆ Understanding developmental plasticity could improve the management of congenital heart defects, where the heart must adapt to abnormal hemodynamics. ◆ This model may help predict long-term outcomes in pediatric patients and guide interventions to optimize cardiac development.
Metabolic adaptations	◆ The neonatal heart primarily relies on glycolysis for energy production, unlike the adult heart, which primarily uses fatty acid oxidation. ◆ Neonatal ABS can reveal how pressure overload affects metabolic pathways during development and whether metabolic shifts contribute to adaptive or maladaptive remodeling.	◆ Insights into neonatal cardiac metabolism could inform therapies for pediatric heart failure, where metabolic dysfunction plays a key role. ◆ This model may also help identify metabolic targets for improving cardiac function in adults with heart failure.
Fibrosis and extracellular matrix remodeling	◆ Neonatal ABS typically results in less fibrosis compared to adult models, suggesting that the neonatal heart has enhanced mechanisms to prevent excessive extracellular matrix deposition. ◆ The composition and turnover of the extracellular matrix may differ in neonates, contributing to their ability to adapt to pressure overload.	◆ Understanding neonatal antifibrotic mechanisms could lead to therapies for reducing fibrosis in adults with pressure overload or heart failure. ◆ This model may help identify biomarkers for early detection of fibrotic remodeling in pediatric patients.
Long-term outcomes	◆ Neonatal ABS can be used to study the long-term effects of early-life pressure overload on cardiac structure and function. ◆ This model may reveal whether early interventions can prevent or mitigate the development of heart disease later in life.	◆ Insights from neonatal ABS could inform strategies for early intervention in pediatric patients with conditions like aortic coarctation or hypertension. ◆ This model may help identify critical windows for therapeutic intervention to prevent long-term cardiovascular complications.

*ABS*, Abdominal aortic banding surgery; *CM*, cardiomyocyte.

## Supplementary Data

Video 1 Illustration of neonatal ABS surgery. Video available at: https://www.jtcvs.org/article/S2666-2507(25)00161-0/fulltext.
