# Supplementary figures and images for: A neonatal rat model of progressive left ventricular pressure overload induced by abdominal aortic banding microsurgery
Source: JTCVS Tech. 2025 Apr 24;32:119–35. doi: 10.1016/j.xjtc.2025.04.014 (PMC12348284; doi:10.1016/j.xjtc.2025.04.014)

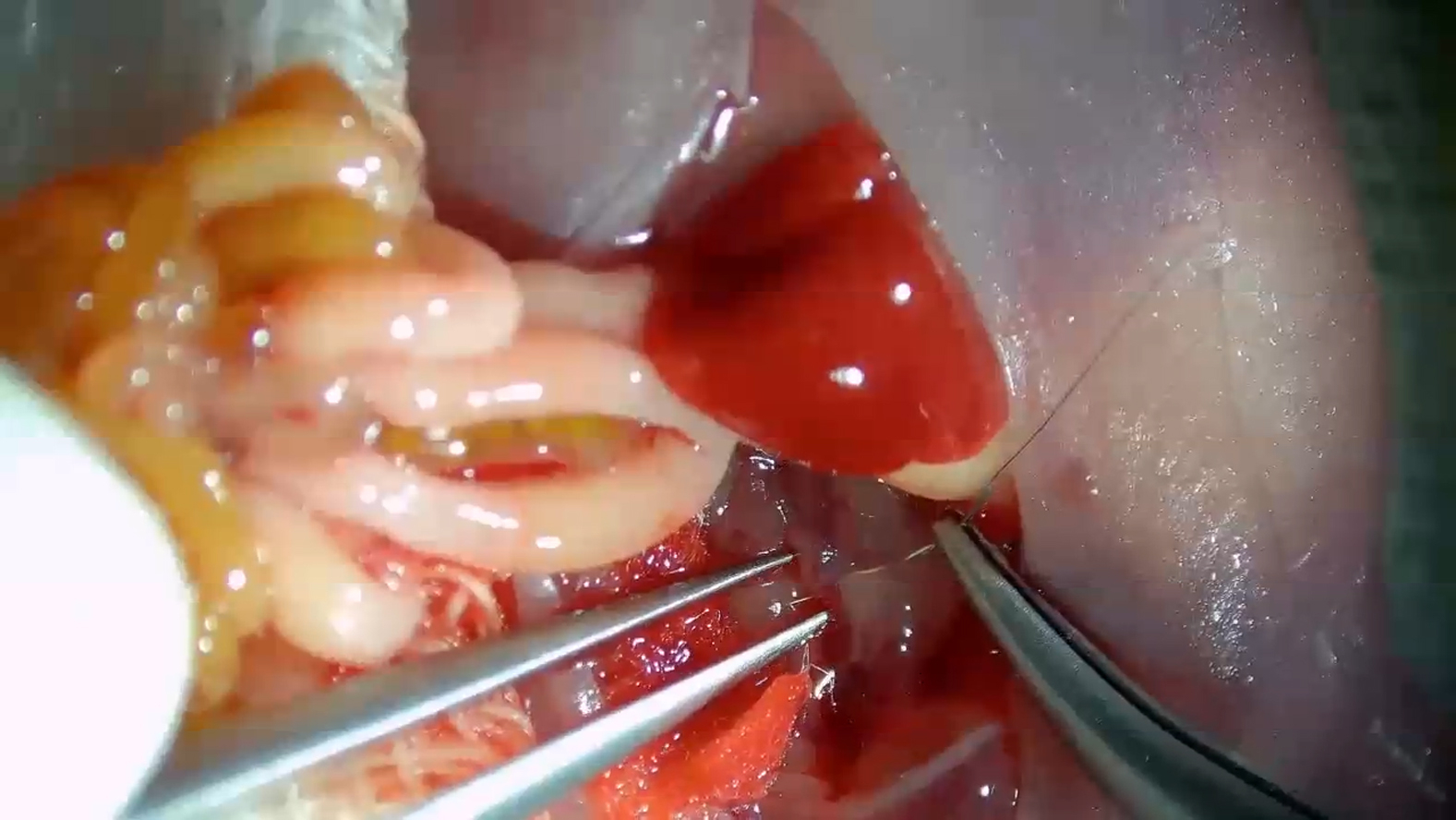

Supplement: Video 1 — Illustration of neonatal ABS surgery. Video available at: https://www.jtcvs.org/article/S2666-2507(25)00161-0/fulltext. [file fx2.jpg]
